# A successful case of deceased-donor liver transplantation from a donor with Marfan syndrome: a case report

**DOI:** 10.1186/s40792-024-01807-y

**Published:** 2024-01-10

**Authors:** Takuma Ishikawa, Shinji Itoh, Takeo Toshima, Yuki Nakayama, Katsuya Toshida, Yuriko Tsutsui, Norifumi Iseda, Takuma Izumi, Shohei Yoshiya, Mizuki Ninomiya, Tomoharu Yoshizumi

**Affiliations:** Department of Surgery and Science, Graduate School of Medical Sciences, Kyushu University, 3-1 Maidashi, Higashi-Ku, Fukuoka-Shi, Fukuoka 812-0054 Japan

**Keywords:** Liver transplantation, Deceased-donor liver transplantation, Marfan syndrome

## Abstract

**Background:**

Liver transplantation is the definitive therapy for patients with decompensated cirrhosis. Marfan syndrome is a systemic inheritable connective tissue disease associated with fibrillin-1 gene mutations, which cause abnormalities in connective tissue. Vascular changes due to Marfan syndrome occur mostly in the main vessels due to the high amount of connective tissue within the vessel wall and the high pressure and blood flow to which they are exposed. The incidence of changes in visceral arteries is about 0.42% and usually presents with cystic medial necrosis. This report is the first deceased-donor liver transplantation with a donor with Marfan syndrome with a history of abdominal surgery.

**Case presentation:**

A patient in his 50s underwent liver transplantation for decompensated alcoholic cirrhosis. The donor, a 50s male with Marfan syndrome, was diagnosed with brain-death due to a cerebral hemorrhage caused by a cerebral aneurysm. The donor’s clinical presentation as Marfan syndrome was aortic dissection, with multiple surgical procedures performed from the aortic root to the abdominal aorta. An intraoperative biopsy of the hepatic artery showed no abnormality, so this organ was considered appropriate. The surgery was completed without any problems of the arterial anastomosis. The patient’s postoperative course was uneventful, and he was transferred to a hospital for recuperation on the 18th postoperative day. One year after the surgery, the patient is still alive without any complications from the transplantation or arterial problems.

**Conclusions:**

Even if the patient had a history of surgery for vascular anomalies extending to the abdominal aorta due to Marfan syndrome, the patient can be a donor for liver transplantation under appropriate judgment, including intraoperative biopsy.

## Introduction

Liver transplantation (LT) is universally performed as a treatment for end-stage liver disease, acute hepatic failure, hepatocellular carcinoma, and several metabolic disorders [1–6]. In Japan, the proportion of deceased-donor liver transplantation (DDLT) in LT is lower than in other countries. As a result, many patients are unable to undergo LT [7].

Marfan syndrome (MS) is an autosomal dominant inheritance of connective tissue disease with an estimated incidence of 1 in 5000; and in 90% of cases, it is caused by mutations in the gene for fibrillin-1, the main component of extracellular microfibrils. The cardiovascular, ocular, and skeletal systems are the main targets of the disease. Early detection and appropriate management are important because patients with MS are prone to life-threatening cardiovascular complications, such as aortic aneurysms and aortic dissection [8]. Therefore, clinical surgeons may often hesitate to perform LT using MS donors in practical terms. However, it was reported that MS cases are likely to have fewer vascular complications in the hepatic artery and other visceral arteries during abdominal surgery [9].

To date there is only one reported case of LT with MS as a donor, and there is little information on whether it is eligible as a donor for LT. This report was first successful liver transplantation donor in a patient with MS with a history of abdominal aortic surgery.

## Case presentation

**Fig. 1:**
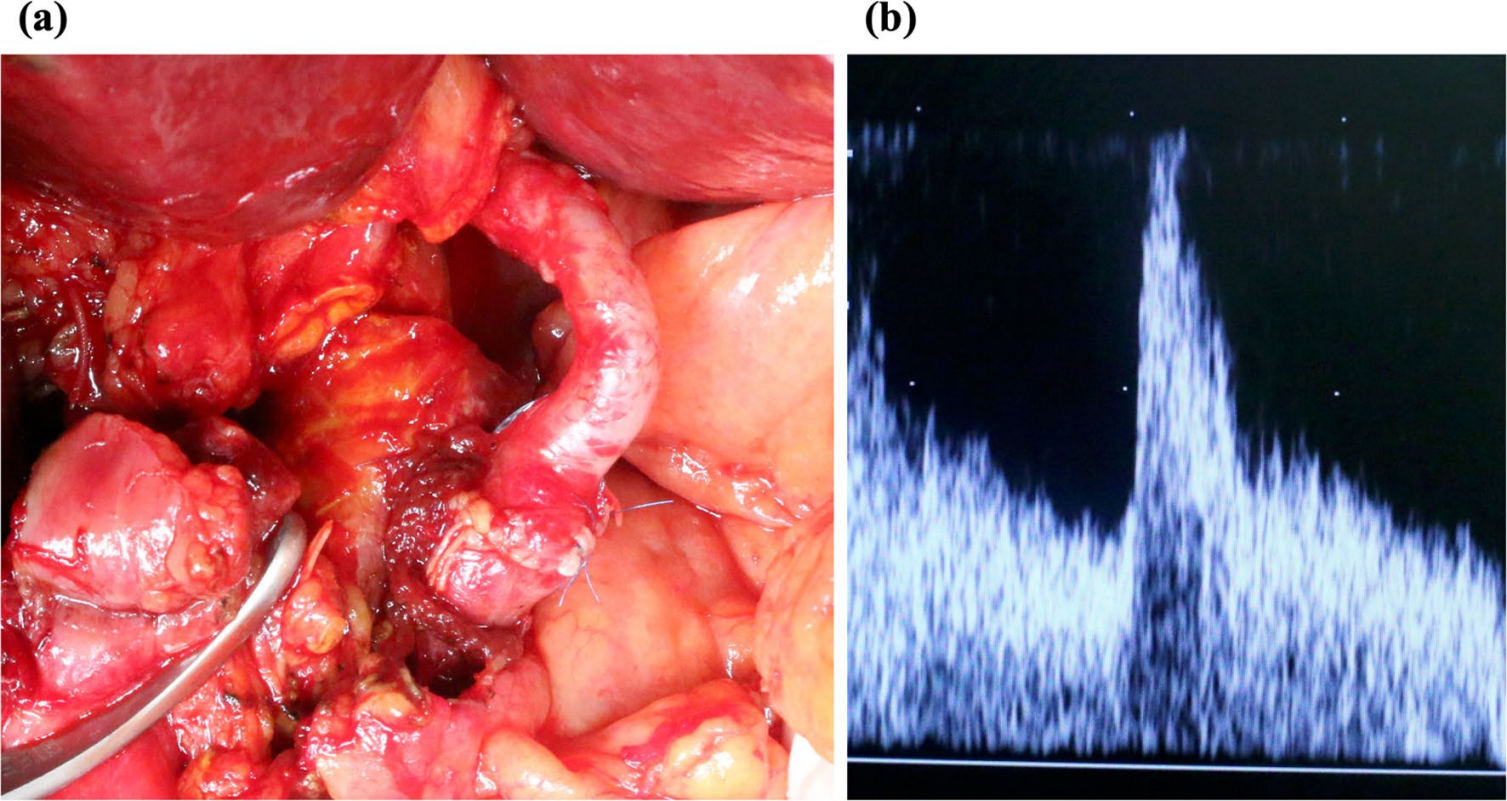
**a** Anastomosis of the recipient’s common hepatic artery with the graft’s common hepatic artery. **b** Evaluation of blood flow in anastomosed arteries by Doppler ultrasound

A male patient in his 50s was on a waiting list for liver transplantation with decompensated alcoholic cirrhosis. The model for end-stage liver disease score was 30, and the Child–Pugh score was 13 (Grade: C). At that time, a brain-dead donor was available. The donor was a man in his 50s and had MS. He had a history of aortic dissection related to MS and multiple surgeries. He had previously undergone total aortic replacement of the ascending arch, aortic root replacement, descending thoracic aortic replacement, abdominal aortic replacement, and aortic aneurysmectomy plus artificial vessel replacement (reconstruction of celiac artery, superior mesenteric artery, bilateral renal arteries). Therefore, during hepatectomy, the liver was removed up to the normal artery that is not artificial blood vessel, and the common hepatic artery was used for reconstruction. Only the liver was procured from this brain-dead donor. Because a biopsy of the hepatic artery was performed during surgery and was normal, this organ was considered suitable. The DDLT procedure proceeded without problems and the arterial anastomosis was completed. The operation duration was 621 min, blood loss was 804 ml, and the type of implantation was conventional, recipient's proper hepatic artery and the donor’s proper hepatic artery were anastomosed with interrupted by using 8-0 Prolene (totally 12 sutures). The postoperative course was uneventful and a daily ultrasound Doppler examination for 2 weeks confirmed that blood flow in the anastomosed vessels was normal. A computed tomography scan 2 weeks after DDLT also confirmed that there were no problems (Fig. 1). The patient was discharged on the 18th postoperative day without major post-implantation complications. One year after surgery, the patient’s systemic condition is still intact.

## Discussion

We experienced DDLT with a donor with MS. DDLT with a donor with MS is rare, with only one case reported in the past (Table 1) [10]. Vascular changes due to MS occur mostly in the main vessels (30–50% incidence). This is associated with the high amount of connective tissue within the vessel wall and the high pressure and blood flow to which the artery is exposed [11–13]. The wall of the aorta is composed of three layers, with a large number of elastin fibers in the tunica media in the middle. The tunica media provides elasticity to the arteries and binds the outer and inner membranes together. In MS, the fibrillin-1 gene is abnormal, which prevents the formation of elastin fibers in the tunica media, making the aortic wall brittle. As a result, aortic aneurysms are more likely to form, and aortic dissection is more likely to occur [14]. When aortic cannulation for perfusion, we need careful cannulation for possible dissection of abdominal aorta because of possible abnormality in the structure of aorta. However, in this case, the common iliac artery had normal elasticity on intraoperative palpation, and the lumen did not feel any change from normal. We think that good perfusion was achieved by careful and careful debridement to avoid divergence and intimal detachment. The incidence of visceral artery changes is approximately 0.4–2.0% and usually manifests with cystic medionecrosis in MS [11–13]. If the biopsy result was strong degeneration with cystic medionecrosis, it is not suitable for anastomosis and liver transplantation may not be performed as planned. The frequency of aneurysms of abdominal viscera is around 0.01–0.2% at autopsy. The patient will continue to undergo computed tomography scan and other imaging evaluations.

**Table 1:** Liver transplantation using a deceased donor with Marfan syndrome

Case	Donor				Recipient		
	Age/sex	MS comorbidities	Cause of brain death	Biopsy of hepatic artery	Age/sex	Etiology of liver disease	MELD
No. 1 (Ref. 10)	10s female	Mitral valve deviation, and osteoarticular abnormalities	Hypoxic encephalopathy	No abnormality	40s male	HCV, HCC	N/A
No. 2 (the present case)	50s male	Aortic dissection	Cerebral hemorrhage	No abnormality	55 male	Decompensated alcoholic cirrhosis	30

HCC: hepatocellular carcinoma, HCV: hepatitis C virus, MELD: model for end-stage liver disease, MS: Marfan syndrome,

N/A: not available

In the literature, only two hepatic artery complications are described in patients with MS [15, 16]. Santiago-Delpin described a case of incomplete MS with multiple aneurysms of the aorta and its branches, in which a hepatic artery aneurysm perforated into the common bile duct. Ruschen published a case in which a spontaneous hepatic artery rupture occurred in a patient with MS.

In the present case, our rationale to accept a donor with MS was based on the low incidence of hepatic artery complications and total absence of hepatic dysfunction in patients with MS. The normal hepatic artery biopsy was also important in the acceptance process. Fibrillin-1 gene mutations persist in the transplanted graft and, theoretically, may increase the risk of vascular complications such as dilation, aneurysmal changes, dissection, and intimal tears. Farese et al. [17] suggested another potential complication occurring in this setting: the possibility of antibodies developed against mutated fibrillin-1 donor liver cells. To date, we have not detected any posttransplant liver dysfunction and the recipient has not presented graft rejection. Three cases of kidney transplantation from MS have been reported. These three patients showed good progress with no postoperative complications [17]. In a case of heart transplantation, Marfanoid aneurysm in donor aorta after transplantation was reported [18]. This report was that the donor has the possibility to be MS due to his physique and other factors, and organ transplantation with main vessels from MS patients may have a certain risks but is feasible.

In summary, a patient with MS and a history of abdominal aortic surgery can still be a donor for DDLT with appropriate judgment. Considering reports of LT using MS donors are infrequent, a biopsy should be performed when transplanting a liver from an MS patient. In addition, a detailed follow-up of the transplanted vessels by Doppler ultrasound is advocated for patients undergoing such transplants [10]. In addition, before organ procurement, recipients should receive adequate informed consent regarding MS. There is no information on the number of DDLT using MS as a donor or their long-term outcome, and this information needs to be gathered in the future.

## Conclusion

We experienced a successful case of DDLT in which a patient with a history of abdominal aortic surgery due to MS was used as a donor. Patients with MS and a history of abdominal aortic surgery can still be donors for DDLT with appropriate judgment based on an intraoperative hepatic artery biopsy.

## Data Availability

The datasets used and/or analyzed during the current study are available from the corresponding author on reasonable request.

## Abbreviations

DDLT: Deceased-donor liver transplantation
LT: Liver transplantation
MELD: Model for end-stage liver disease
MS: Marfan syndrome

## References

[1] Yoshizumi T, Itoh S, Shimokawa M, Inokuchi S, Harada N, Takeishi K (2021). Simultaneous splenectomy improves outcomes after adult living donor liver transplantation. J Hepatol.

[2] Yoshizumi T, Ikegami T, Kimura K, Uchiyama H, Ikeda T, Shirabe K (2014). Selection of a right posterior sector graft for living donor liver transplantation. Liver Transplant.

[3] Toshima T, Yoshizumi T, Kosai-Fujimoto Y, Inokuchi S, Yoshiya S, Takeishi K (2020). Prognostic impact of osteopenia in patients who underwent living donor liver transplantation for hepatocellular carcinoma. World J Surg.

[4] Toshima T, Ikegami T, Kimura K, Harimoto N, Yamashita Y, Yoshizumi T (2014). Application of postoperative model for end-stage liver disease scoring system for evaluating liver graft function after living donor liver transplantation. Transplant Proc.

[5] Yoshiya S, Yoshizumi T, Iseda N, Takeishi K, Toshima T, Nagao Y (2020). Anastomosis of the common hepatic artery and round ligament as portal vein arterialization for hepatic artery occlusion after deceased donor liver transplantation: a case report. Transplant Proc.

[6] Rawal N, Yazigi N (2017). Pediatric liver transplantation. Pediatr Clin N Am.

[7] Yoshizumi T, Itoh S, Imai D, Ikegami T, Ninomiya M, Iguchi T (2015). Impact of platelets and serotonin on liver regeneration after living donor hepatectomy. Transplant Proc.

[8] Coelho SG, Almeida AG (2020). Síndrome de Marfan revisitada—da genética à clínica. Rev Port Cardiol.

[9] Grotemeyer D, Duran M, Park EJ, Hoffmann N, Blondin D, Iskandar F (2009). Visceral artery aneurysms—follow-up of 23 patients with 31 aneurysms after surgical or interventional therapy. Langenbeck’s Archives Surg.

[10] Benedetto FD, Sandro SD, Ruvo ND, Masetti M, Quintini C, Montalti R (2007). Liver transplantation from a donor affected by Marfan’s syndrome. Transplantation.

[11] Judge DP, Dietz HC (2005). Marfan’s syndrome. Lancet.

[12] Dietz HC, Cutting CR, Pyeritz RE, Maslen CL, Sakai LY, Corson GM (1991). Marfan syndrome caused by a recurrent de novo missense mutation in the fibrillin gene. Nature.

[13] Arneson MA, Smith RS (2005). Ruptured hepatic artery aneurysm: case report and review of literature. Ann Vasc Surg.

[14] Halper J, Kjaer M (2013). Progress in heritable soft connective tissue diseases. Adv Exp Med Biol.

[15] Santiago-Delpin EA, Marquez E, Rodriguez OL, Oliveras FE, Baldizon C, Martinez-Cabruja R (1972). Perforated hepatic artery aneurysm and multiple aneurysms in incomplete Marfan syndrome. Ann Surg.

[16] Ruschen B, Hamann HJ (1984). Rupture of the common hepatic artery in Marfan syndrome. Zbl Chir.

[17] Farese S, Vogt B, Frey FJ, Huynh-Do U (2006). Successful kidney transplantation from Donor with Marfan’s syndrome. Am J Transplant.

[18] Pathi VL, Pillay TM, Wheatley DJ, Belcher PR, Naik SK (1997). Marfanoid aneurysm in donor aorta after transplantation. Ann Thorac Surg.

